# Dynamic Behavior of Engineered Lattice Materials

**DOI:** 10.1038/srep28094

**Published:** 2016-06-20

**Authors:** J. A. Hawreliak, J. Lind, B. Maddox, M. Barham, M. Messner, N. Barton, B. J. Jensen, M. Kumar

**Affiliations:** 1 Lawrence Livermore National Laboratory, Livermore, CA 94550 USA; 2 Institute for Shock Physics, Washington State University, Pullman, WA 99164 USA; 3 Los Alamos National Laboratory, Los Alamos, NM 87545 USA

## Abstract

Additive manufacturing (AM) is enabling the fabrication of materials with engineered lattice structures at the micron scale. These mesoscopic structures fall between the length scale associated with the organization of atoms and the scale at which macroscopic structures are constructed. Dynamic compression experiments were performed to study the emergence of behavior owing to the lattice periodicity in AM materials on length scales that approach a single unit cell. For the lattice structures, both bend and stretch dominated, elastic deflection of the structure was observed ahead of the compaction of the lattice, while no elastic deformation was observed to precede the compaction in a stochastic, random structure. The material showed lattice characteristics in the elastic response of the material, while the compaction was consistent with a model for compression of porous media. The experimental observations made on arrays of 4 × 4 × 6 lattice unit cells show excellent agreement with elastic wave velocity calculations for an infinite periodic lattice, as determined by Bloch wave analysis, and finite element simulations.

The physical world is filled with cellular structures – both man made, like truss bridges, and of natural origin, like porous bone tissue. These entities, along with many other examples, large and small, are demonstration of a material response being optimized through topology rather than composition. Cellular materials have received significant attention over the last several decades for their thermal^1^, electrical^2^, and optical properties^3^,^4^ as well as for their potential as lighter weight replacements for bulk materials^5^. Conventional materials design relies on manipulation of chemistry or phase fractions (for multi-phase systems) or microstructural length scales, such as grain size distributions^6^,^7^. However, with the advent of additive manufacturing (AM) it is now possible to manipulate the architecture of the cellular material to impart order and periodicity into materials at the mesoscopic scale and go beyond the notion of simply seeding random porosity. The ability to organize the structure and composition at the mesoscopic scale allows the custom design of materials by applying structural engineering principles, like truss theory, at the micron length scale. This will open up the possibility of designing and engineering material properties to precisely meet the demands of the intended application^8^.

Recent reports in the literature have shown impressive mechanical properties for such architected materials^8^. These measurements were performed on bulk samples and do not address the fundamental length scales associated with the emergence of continuum behavior in a material that is more open space than solid. These measurements are also exclusively in the realm of quasi-static loading, where the time dependent properties that pertain to energy absorption under high velocity ballistic impact or application to high speed devices, are not probed.

Dynamic loading conditions are characterized by large gradients in stress and strain where the length scale of these gradients is smaller than the engineered lattice unit cell size. For instance, it is easy to formulate ideas of stationary waves of a high amplitiude traveling through crystal lattices, which have long range periodicity but the length scale (typically on the order of Angstroms in high symmetry structures such as a face centered cubic lattice) is much shorter than that of the traveling wave. It is generally agreed that a stochastic foam with cell sizes on the order of a few nanometers only supports a compaction wave.

So the open question is the nature of a compression wave in long-range periodic lattice structures with a length scale comparable to the wavelength of the traveling wave. Such a lattice structure has characteristics of both porous media as well as crystal lattices. Lattice characteristics are described by collective behavior with directional dependencies in the response while in porous media the response is only related to the initial density^9^. A better understanding of wave propagation, achieved through dynamic experiments, is critical to determining the mechanical response of lattice materials, and will elucidate fundamental properties of the time-dependent response of this new class of materials. Of particular interest is when the properties of the imposed mesoscopic, long range periodic lattice begin to emerge, versus the discrete behavior of the individual structures in a lattice cell.

*In situ* x-ray phase contrast imaging was used to directly measure the response of mesoscopic engineered lattice structures under impact loading^10^,^11^. We investigate the time-dependent response of octet truss (stretch-dominated) structures compressed along three different structural axes, as well as a Kelvin cell (bend-dominated) and a stochastic, random structure compressed along a single axis, to compare the response of structured versus non-structured materials. The impact provides a near instantaneous mechanical impulse to a sample surface from which the material compaction and elastic properties as a function of topology can be measured.

In this article we investigate the coordinated behavior in the dynamic properties of engineered lattice structures using shock loading. The investigation looked at two properties. The first relates to the compaction of the lattice, which is characterized by a large change in density with the lattice structure breaking and collapsing. The second is the elastic properties associated with an initial compressive distortion of the lattice. Even through the samples are only 4 × 4 × 6 unit cells in size, we observe collective behavior in both the compaction and elastic dynamic properties of the lattice materials immediately after impact.

## AM Structures

**Figure 1:**
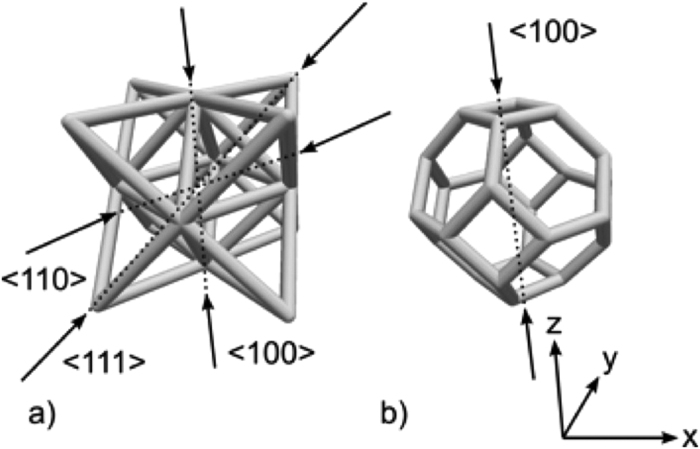
(**a**) Octet truss unit cell; (**b**) Kelvin cell unit cell. Arrows indicate experimental impact

The test samples were fabricated with two different engineered lattice architectures having the same bulk density, Fig. 1. The microlattices were produced by projection microstereolithography, a layer-by-layer additive micromanufacturing process capable of fabricating arbitrary three-dimensional microscale structures from 1,6-hexanedol diacrylate (HDDA)^12^. One engineered lattice sample had a face-centered cubic (FCC) octet topology, Fig. 1a). This structure has a stretch-dominated response, in which deformation primary occurs via axial tension and compression in the struts^13^. The nodes of the octahedron fall on the center of the unit cell, while the struts of the corner tetrahedron are shared between adjacent unit cells. The cubic symmetry of the lattice engenders approximately isotropic behavior. The octet structure has a high stiffness-to-weight ratio attributed to the struts carrying load under compression or tension rather than bending. The octet structure materials were impacted along the three principal axes of the unit cell, namely <100>, <110>, and <111> (see Fig. 1a).

The second structure was a Kelvin cell, the tetrakaidecahedral structure first calculated by Lord Kelvin to describe the shape of infinitely interacting bubbles of the same volume and pressure^14^, Fig. 1b). Like the octet cell, the elastic properties of the Kelvin cell are approximately isotropic. However, unlike the octet, the strength of the Kelvin structure relies on the bending stiffness of its members.

The results from these lattice structures were compared with that of a stochastic foam, where the ligament length-scale and bulk density were designed to match the structured lattice cells, but with no ordered topology. For all structures the diameters of the struts were 30 μm and sample dimensions were 1.2 × 1.2 × 1.8 mm with 300 μm unit cell size. For example, the octet <100> sample had 4 × 4 × 6 unit cells.

### Description of Experiments

**Figure 2:**
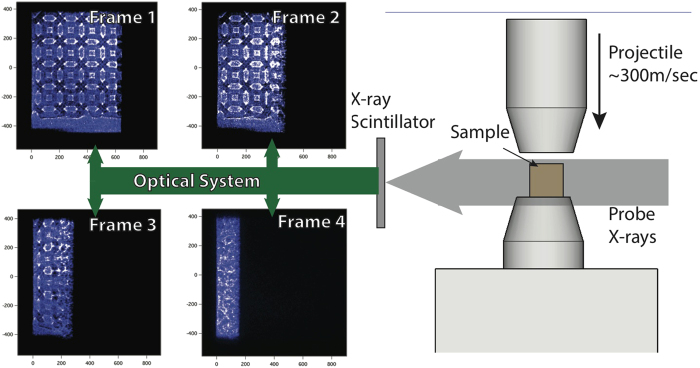
A schematic diagram of the experimental setup with 4 example frames from a single Kelvin foam experiment. The x-rays probe parallel to the impact face.

Phase contrast imaging (PCI) of AM samples subject to dynamic loading was performed at the Dynamic Compression Sector (DCS) (Sector 35) at the Advanced Photon Source (APS) of the Argonne National Laboratory (ANL)^10^,^11^. Dynamic loading using the IMPULSE gas gun system^15^ provides a nearly instantaneous velocity rise at the sample surface from which the time-dependent response of the trusses within the sample can be measured using PCI. A series of four 12 keV x-rays images were taken in a side-on geometry, normal to the impact direction, in multiples of 153.4 nanoseconds. Images were captured on a LSO scintillator and optically relayed to four gated CCD detectors (PI-MAX II) that provide the single pulse isolation^11^. Figure 2 shows a schematic diagram of the target configuration with sample data from the Kelvin foam.

The experiments were all conducted with an impact velocity tuned to be 315 m/s using aluminum projectiles. The projectiles were designed with a 6 mm long, 3 mm diameter anvil on the leading edge to minimize view obstruction due to any minor misalignment of the gun axis. Piezoelectric (PZT) pins attached to the target block were used to measure the time of impact with the gun projectile to synchronize the imaging system with the sample impact. The projectile velocity was measured with photo Doppler velocimetry (PDV) using a collimating fiber optic probe (AC Photonics) mounted next to the sample.

## Experimental Results

Dynamic loading of the samples through impact provides a fiducial (at the time of the impact) from which the time-dependent response of the material can be measured. Similar to quasi-static experiments, the boundary condition on the sample provides a single loading direction, and the resulting material response can be measured. Unlike quasi-static measurements, the stress waves under dynamic loading do not have time to traverse the sample and equilibrate before the measurements are made. Consequently, large stress and strain gradients occur in the dynamic experiments. There are two distinct material responses observed in the PCI images. After impact, an elastic disturbance propagates through the lattice, leaving the lattice structure intact and making minimal changes to the density. This elastic response is followed by a densification front that advances more slowly from the impact plane, disrupting the lattice structure and significantly increasing the density of the material.

**Figure 3:**
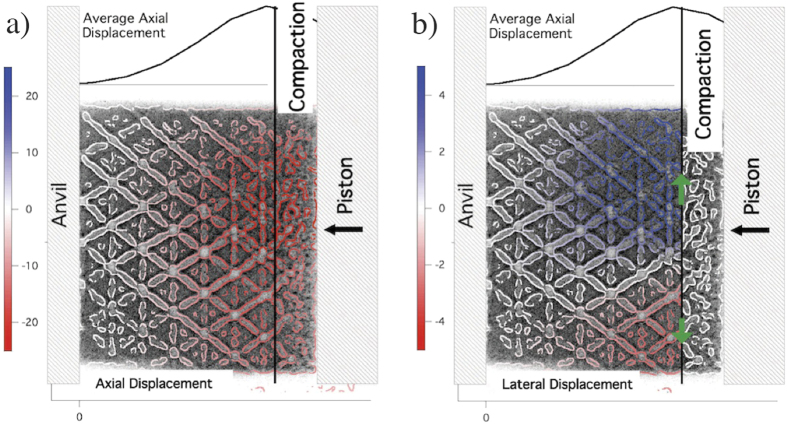
A color overlay showing the (**a**) axial and (**b**) lateral displacements of the lattice based on point to point displacement analysis. The positive and negative numbers correspond to the image direction, where the compaction is in the negative direction. The lateral displacement is small and consistent with stress relief at the open surfaces.

The signature of the densification front and compaction region behind it can readily be seen in the experimental images. The compaction region is where the ligaments in the structure have collapsed and the overall density is higher than in the initial undeformed structure. At this point the density within the deformed state acquires a terminal value with no further relative motion within it. A vertical black line in Fig. 3 demarcates the compacted region on the impact (piston) side from the undeformed region on the anvil side. The local density behind and in the compaction region differ significantly over the short distance that is the densification front. We define the densification front as the point where the measured local density is halfway between those two measured densities, namely the undeformed density and the compacted density.

Unlike the compaction region, the signature of the elastic front cannot readily be seen in the experimental images. We can infer the elastic wave has traversed through the sample material if the local materials points, such as the nodes, have moved an amount that is within the experimental sensitivity. The sensitivity turns out to be ~4μm, which is to say that on the leading edge of the elastic front, the local material will have moved less than 3 pixels in the experiment images. The propagation of this wave cannot be seen by eye in the sequence of images show in Fig. 2, but Fig. 3 highlights the local axial and lateral displacements as is calculated by a point-to-point comparison of images method described later.

### Compaction

**Figure 4:**
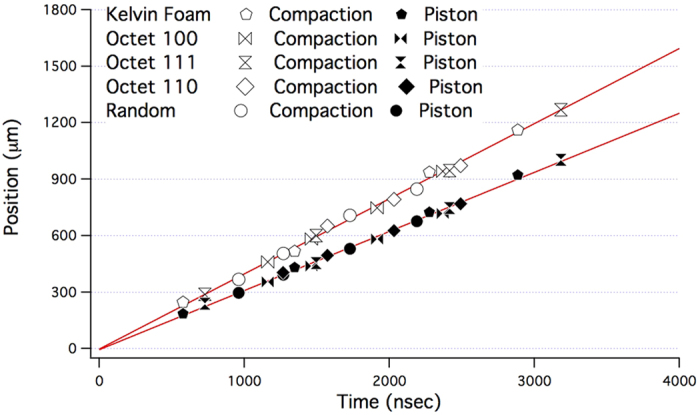
Plotting the sample impactor interface and compaction front position as a function of time for all engineered lattice materials, normalized such that t = 0 corresponds to surface impact and x = 0 to the impact surface.

Figure 4 is a plot of the position of the impactor/sample interface and the leading edge of the compaction wave as a function of time. All samples have their origins set such that the impact surface and impact time are defined as x = 0 and t = 0, respectively. The leading edge of the densification front is taken as the point where the material density most sharply rises from that of the undeformed lattice when looking from the anvil towards the projectile. Using the known time differences between frames of the experiment, one can calculate the compaction wave velocity as v_c_ = Δx_c_/Δt. This is just the distance the densification front moves divided by the inter-frame time. Figure 4 indicates the position of the densification front with a solid black line at this particular frame. Figure 2 also shows the progression of the compaction wave without specifically indicating its position. The 3% variation in the initial impact velocity is smaller than the uncertainity of the impactor and compaction wave velocity using the position in the image and timing of the frames. For the compaction wave, all material structures show the same velocity to within experimental uncertainties, with the impactor travelling at 314 ± 6 m/s and the compaction front moving at 399 ± 25 m/s, with a 6% systematic uncertainity in the absolute velocity due to the calibration of the magnification of the imaging system.

Over the timescale of the experiment the compaction front speeds are constant, suggesting the formation of a steady wave. The position axes are marked in 300 μm increments, the nominal unit cell size for the AM structures considered in this study. We observe that a steady wave profile forms over less than a single engineered lattice unit cell. This is important because this wave does not appear to be sensitive to the local density fluctuations in the lattice unit cell yet still exhibits a collective response over the length of a single lattice cell. The independence of the compaction wave speed to the initial lattice structure suggests that the compaction response maybe related simply to density as described by porous Hugoniot models, which do not take into account the actual cellular size and compaction front width. This observation suggests that a porosity model could be used to determine compaction, ignoring the initial lattice structure since the steady wave develops very rapidly after impact.

Using the steady-wave approximation and conservation of mass, the compacted material exhibits 4.6 times the intial density. Since the initial density was 10% solid density, the final average density is still 46% of solid HDDA density in the compaction region, demonstrating the material still has some strength to support a structure — a zero strength material would collapse to 100% ambient solid density. Assuming a steady wave and using the Rankine Hugniot relations, the longitudinal stress at the aluminum sample interface can be estimated as 12.5 MPa. Based on impedence calculations this longitudinal stress at the interface would slow the aluminum impactor by 2 m/s, Since this is smaller than the velocity uncertainity of our imaging experiment it is not possible to determine the resulting stress from the AM lattice material on the impactor.

### Elastic Properties

The Bloch wave theorem can be used to calculate the dynamic response of a periodic structure as the composition of shifted plane waves over a single unit cell^16^. Using the theorem one can construct the dispersion relation – the relation between wave vector and frequency – for a periodic octet lattice from the dynamics of a single octet unit cell. In the limit of long wavelength, the Bloch wave theorem degenerates to an equivalent continuum theory with the inertial force relation corrected for micro-inertial effects^17^. The propagation of the displacement due to a compaction wave is carried by the zero frequency term, *i.e*., the long wavelength component. In this limit an elastic disturbance will propagate at a fixed speed independent of wavelength. In the experiments here as the samples are short, relative to the unit cell size, we can compare the elastic wave speed to the single value given by the long wavelength limit, assuming dispersion can be neglected at these short length scales.

The elastic displacement is measured using a point-to-point comparison technique of images of the sample taken before and during the experiment of the material ahead of the compaction wave. Figure 3 shows an image of an octet truss impacted along the <100> direction, in the axial direction along the compaction direction and lateral to the shock direction. Figure 3a) shows the longitudinal displacement along the axial direction, which is fairly uniform across the sample with slight reductions in magnitude at the edges of the sample. This reduction is most likely due to edge effects and can be ignored. The lattice displacement is at a maximum at the compaction front, denoted by the black line in Fig. 3a) and to first approximation decreases linearly towards the anvil. This suggests that the strain can be given by a single value given by the gradient in the displacement 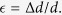
Figure 3b shows the lateral displacement that appears to be symmetric about the axis of the sample, which is consistent with effects of the edge of the sample. The strain is calculated using a linear fit to the longitudinal lattice displacement. The leading edge of the elastic strain wave is taken as the point where the fitted displacement goes to zero. Using the time difference between the different frames of the experiment we can now infer the elastic wave velocity.

Within the resolution of the measurement, the lattice shows the fast elastic wave travelling at a speed of 935 ± 100 m/s in the <001> direction, with a characteristic elastic strain of 3.4 ± 0.5%. The <110> and <111> orientations show elastic waves speeds of 1050 ± 100 m/s and 1179 ± 100 m/s, respectively, with 2.5 ± 0.5% and 4.5 ± 0.5% elastic strain, respectively. The Kelvin cell shows a slower elastic wave speed at 715 ± 100 m/s with a 3.5 ± 0.5% elastic strain. The elastic wave speed is taken on the central axis of the sample to minimize the boundary affects associated with release from the edges. The observation of an elastic disturbance in the lattice structure shows coordinated behavior and lattice theory can be used to describe the elastic properties even though the elastic strain has not had a chance to traverse the sample.

For stretch-dominated lattice materials two parameters, the Young’s modulus of the material of the struts and the density of the lattice, determine the response of the equivalent continuum. The value of strain supported by the lattice is assumed to correspond to the Hugoniot elastic limit (HEL) before lattice failure and compaction occur and requires supplementing the elastic model with a yield surface. Messner *et al*. describe how to construct an effective yield surface for a stretch dominated lattice^17^,^18^. Table 1 summarizes the speeds generated by this procedure for the octet lattice using a Young’s modulus and density typical for the material (see Table 2). For the different orientations of the octet truss structure this model, including the micro-inertial effects, predicts wave speeds that approximately match those measured in the experiments, Table 1. The Bloch wave theorem assumes an infinite periodic structure with no consideration for timescale, *i.e*., effectively infinite time. The agreement with the elastic wave speeds in the experiment suggest that the continuum model for the elastic properties of these structures can also be used predict the elastic properties of the dynamic response.

### Simulations

Finite element simulations of the octet truss structure were conducted in ALE3D, an arbitrary Lagrangian-Eulerian finite element package developed by Lawrence Livermore National Laboratory^19^. The simulations use periodic boundary conditions and the material properties used for HDDA in the simulations are listed in Table 2. Similar to the experiments, the simulated compression waves in all the three octet lattice structures develop the same two-front behavior; a faster front associated with elastic deformation and a slower front associated with compaction.

**Figure 5:**
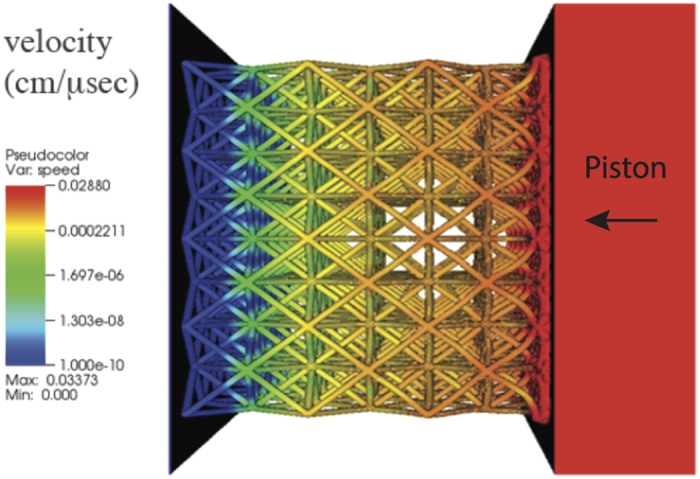
The finite element simulation where the color scale indicated the material velocity. Note: the colors are on a log scale.

Figure 5 shows the simulation of the <100>-orientated octet lattice at a time after impact, when the two fronts have separated. The fringe colors show the material velocity in the *x*-direction. The sharp increase in material velocity well ahead of the impactor indicates the elastic front. Table 1 lists the speed of the elastic front and the magnitude of the associated elastic strain in each simulation. The computed speeds from simulation and theory closely match the speeds observed in the experiments.

In the simulation the elastic front amplitude is observed to decay and the front speed decreases over time in simulations of much longer lattices than the samples used in the experiments. This suggests the lattice topology may cause attenuation and dispersion that affects the elastic wave. The samples used in the experiment are the largest currently able to be probed, thus limiting our ability to explore the true nature of the waves formed under dynamic compression. Further development of the experimental technique is required to probe larger samples to detect any dispersion in the elastic wave.

## Discussion

The dynamic response of additively manufactured engineered lattice (cellular) materials was investigated using *in situ* x-ray phase contrast imaging. The time resolution afforded by high photon flux synchrotron sources enabled us to directly capture the traveling waves through the underlying lattice architecture, while the fidelity of the data allowed quantification of this interaction to a degree hitherto not reported. Three different structures were impacted at ~315 m/s and compaction and elastic deformation were observed.

It was found that within the resolution of the experiment the compaction of the lattice is steady and is established on a length scale that is a fraction of the engineered lattice unit cell, which is on the order of 300 μm. The compacted density is insensitive to the initial material structure and, within the parameters of the experiment, has sufficient strength to maintain a density that is 46% that of fully dense HDDA. Suggesting for these materials the compaction of the material can be described by porous material models.

The elastic properties of the engineered lattice, as predicted by the Bloch wave theory, can be used to accurately determine the elastic response of the periodic lattices, even through the theory assumes an infinite periodic structure and the sample size is only a few unit cells. Elastic disturbances in the stochastic foams were not observed within the resolution limits of our experiments. Remarkably, the expected anisotropic character of wave propagation in the cubic-symmetry lattices was also captured, which suggests additional possibilities that are available to the materials designer as they consider various lattice types for specialized applications.

The observation (and an understanding) of collective behavior on short length and fast time scales is critical to building accurate models of the response of AM structured materials. The basic approach is to be able to use the collective properties of an engineered structure at the micron scale to influence the behavior of a material. The key aspect is to gain an understanding of when the collective properties of the impose structure emerge. Conventional thought would suggest that collective properties only emerge in applications where the length scale of interest is large compared to the lattice length scale. Observations show that the elastic and compaction behavior exhibits collective response rapidly under shock loading. The material response can be treat as classical elastic-plastic deformation even though the stress and strain gradients are much smaller then a unit cell.

To further test the applicability of the Bloch theorem to the elastic response of the AM lattice materials, it would be important to perform experiments on larger samples. Larger samples would be used to investigate dispersion and attenuation of the elastic wave through the material and the steadiness of the compaction wave. Further experiments could be conducted with varying parameters such as truss thickness and unit cell size, which could test elastic theory and whether compaction follows a porosity-based model. With near infinite variability in topological configurations shock loading experiments represent a technique for a fundamental approach to understanding the dynamic properties of AM lattice materials.

## Additional Information

**How to cite this article**: Hawreliak, J. A. *et al*. Dynamic Behavior of Engineered Lattice Materials. *Sci. Rep.* **6**, 28094; doi: 10.1038/srep28094 (2016).

## Figures and Tables

**Table 1:** Comparison of the front speeds in the octet lattice between the experiments, the theoretical predictions, and the finite element simulations for three orientations.

	Elastic Speed (m/s)			Elastic Strain		
	Experiment	Simulation	Theory	Experiment	Simulation	Theory
Octet [100]	935 ± 100	989 ± 59	897	3.4 ± 0.5%	2.5 ± 0.7%	3.8%
Octet [110]	1050 ± 100	1391 ± 155	975	2.5 ± 0.5%	1.5 ± 0.7%	1.5%
Octet [111]	1179 ± 100	1228 ± 100	1094	4.5 ± 1.0%	2.0 ± 0.9%	2.0%

**Table 2:** Material properties for the FE simulations.

Quantity	Description	Value
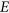	Young’s modulus	1780 MPa
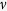	Poisson’s ratio	0.35
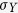	Yield stress	40 MPa
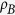	Material density	1.18 g/cm^3^

The material is perfectly plastic post-yield. The relative density of each simulation is calibrated to match the corresponding experimental samples.
